# Intracoronary and peripheral blood levels of TNF-like Cytokine 1A (TL1A) in patients with acute coronary syndrome

**DOI:** 10.1097/MD.0000000000020305

**Published:** 2020-05-29

**Authors:** Xinjing Chen, Yansong Guo, Li Lai, Shengli Zhang, Zhiliang Li

**Affiliations:** a Department of Cardiology, Zhujiang Hospital, Southern Medical University, Guangzhou, Guangdong; b Department of Cardiology; c Fujian Key Laboratory of Cardiovascular Disease, Fujian Provincial Hospital, Fujian Medical University; d School of Humanities and Management, Fujian University of Traditional Chinese Medicine, Fuzhou, China.

**Keywords:** acute coronary syndrome, main adverse cardiac event, TNF-like cytokine 1A

## Abstract

**Background:**

TNF-like cytokine 1A (TL1A) is a subgroup of the tumor necrosis factor superfamily that exerts pleiotropic effects on cell proliferation, inflammation, activation, and differentiation of immune cells. The purpose of the current study is to investigate the clinical significance of TL1A expression in coronary and peripheral blood of patients with acute coronary syndrome (ACS) to determine if TL1A levels can serve as an accurate prognostic indicator.

**Methods:**

A total of 141 patients undergoing coronary angiography were divided into 4 groups: Control (n = 35), Unstable Angina (UA) (n = 35), acute non-ST segment elevation myocardial infarction (NSTEMI) (n = 37), and acute ST segment elevation myocardial infarction (STEMI) (n = 34). The levels of TL1A, MPO, hs-CRP, and IL-10 were detected in coronary and peripheral blood using enzyme linked immunosorbent assay (ELISA), and the MACE incidence rates were compared during 26.3 months of follow-up.

**Results:**

TL1A levels were not significantly different between the UA group and control group. In the UA group, TL1A levels were not significantly different between coronary blood and peripheral blood. However, TL1A levels were higher in the STEMI and NSTEMI groups than in the control group (*P* < .05). Moreover, TL1A levels in the coronary blood of the STEMI and NSTEMI groups were higher than in the peripheral blood (*P* < .05). The expression of TL1A in the coronary blood was the highest in the STEMI group. In addition, TL1A level in the coronary blood was highly correlated with levels in the peripheral blood (correlation coefficient: 0.899, *P* < .001). The hs-CRP and MPO levels in the coronary and peripheral blood of all the UA, NSTEMI, and STEMI groups were higher than the control group. Plasma IL-10 levels in all the UA, NSTEMI and STEMI groups were lower than those in the control group. Plasma TL1A level was positively correlated with the cTnI level, degree of coronary thrombus burden, occurrence of slow coronary flow / no coronary reflow and MACE, but negatively correlated with the IL-10 level or non-correlated with the Syntax score.

**Conclusion:**

Plasma TL1A concentration levels can be used as a predictor of inflammatory response and prognosis in patients with ACS.

Trial Registration: ClinicalTrials.gov, number: NCT02430025; Unique Protocol ID: FJPH20150101; Brief Title: Fujian Province Cardiovascular Diseases Study (FJCVD)

## 1 Introduction

Acute coronary syndrome (ACS) is a broad classification used to describe coronary artery atherosclerosis lesions, which can be caused by a large number of factors including vascular stenosis, occlusion, myocardial ischemia, hypoxia, necrosis, inflammation, and apoptosis of myocardial cells.^[1,2]^ Research has suggested that inflammation and apoptosis contribute to the initiation and development of ACS, which are considered accurate prognostic indicators.^[3,4]^

TNF-like cytokine 1A (TL1A) is a new subgroup of the tumor necrosis factor superfamily (TNFSF).^[5]^ TL1A exerts pleiotropic effects on cell proliferation, activation, and differentiation of immune cells (including helper T cells and regulatory T cells). It not only can induce apoptosis and activate NF-ΚB, but also plays an important role in the activation and proliferation of lymphocytes, monocytes / macrophages, regulation of the inflammatory and immune responses of the body,^[6,7]^ and occurrence and development of atherosclerosis^[8]^ and other diseases.

Recent research carried out by Kang revealed elevated levels of TL1A expression in regions rich in macrophage/foam cells.^[9]^ Treatment of THP-1 cells with recombinant TL1A in combination with IFN-γ also caused induction of MMP-9 and IL-8. These findings suggest that TL1A is involved in atherosclerosis via the induction of pro-inflammatory cytokines/chemokines and decreasing plaque stability by inducing extracellular matrix degrading enzymes.^[9,10]^

The clinical significance of TL1A expression levels in coronary and peripheral blood of ACS has never been reported. In this study, TL1A plasma concentration levels (intracoronary and peripheral blood) were investigated in clinical patients with ACS to evaluate the correlation between TL1A levels and MACE through 26.3 months of follow up. This pilot study was therefore designed to test the hypothesis that TL1A may predict major adverse cardiovascular events.

## 2 Materials and methods

### 2.1 Study population and ethics statement

From February 2013 to January 2014, a total of 141 patients undergoing coronary angiography admitted to the Department of Cardiology, Fujian Provincial Hospital were chosen and divided into 4 groups: the acute ST segment elevation myocardial infarction (STEMI) group, who received emergency percutaneous coronary intervention (PCI) (n = 34), acute non-ST segment elevation myocardial infarction (NSTEMI) group, who received emergency PCI (n = 37), unstable angina (UA) group (n = 35) and control group (n = 35) according to the WHO criteria for diagnosis of coronary heart disease. Patients with vascular diameter stenosis of < 50% (confirmed by coronary angiography and excluded for having coronary artery spasm) were enrolled in the control group.

In accordance with any 2 of the following three items, the diagnostic criteria for acute myocardial infarction (AMI) were: persistent severe chest pain for over 30 minutes, typical dynamic changes in ECG, and increased markers of myocardial necrosis. ST segment elevation in two adjacent leads shown on the ECG was considered acute ST elevation myocardial infarction, while ST segment depression was considered acute non-ST elevation myocardial infarction.

The diagnostic criteria for UA include changes in inducement, location, nature, duration, accompanying symptoms, and remission of ischemic chest pain occurred in patients within 1 to 3 months before admission; transient horizontal or downsloping ST segment depression of 1 mm with or without T-wave inversion; no increased myocardial enzymes or myocardial necrosis markers; and coronary angiography diameter stenosis of at least one vessel being >50%.

The exclusion criteria included the presence of any inflammatory disease, autoimmune disease, heart valve disease, liver and kidney disease, malignant tumors, and blood system disease.

This study was approved by our Ethics Committee before all subjects signed informed consent forms.

For patients with acute coronary symptom (ACS) undergoing PCI was established according to *Chinese Guideline for Percutaneous Coronary Intervention (2016)*.^[11]^ The emergency PCI refers to the patients undergoing PCI within 12 hours after diagnosis of myocardial infarction for acute chest pain.

### 2.2 Coronary angiography analysis

All patients received dual antiplatelet therapy (DAPT) before coronary angiography using the standard Judkins method by placing the catheter via femoral or radial artery. The Artis Zee Floor Eco (an angiography machine manufactured by SIEMENS) was used to analyze the coronary angiography images. Coronary artery disease (CAD) is defined as coronary artery diameter stenosis of ≥50% as indicated by coronary arteriography, which should be determined by independent analysis by at least 2 physicians who have been engaged in coronary intervention for more than 10 years.

### 2.3 Syntax score

A Syntax score for patients with ACS was assigned according to a previous study^[12]^ using healthy volunteers as control. TL1A levels were compared and the correlation of TL1A with Syntax score was analyzed.

### 2.4 Collection of peripheral and coronary blood samples

The radial or femoral artery was routinely punctured before coronary angiography, after which a sheath tube was placed and blood was drawn immediately, placed in an EDTA anticoagulant tube, and then stored in a 4°C refrigerator immediately. The successfully collected peripheral blood flowed via the arterial sheath tube from the angiographic catheter to the coronary artery orifice. After confirming that the angiographic catheter was correctly placed in the coronary artery, 10 ml of blood was taken and placed in another EDTA anticoagulant tube and then stored in a 4°C refrigerator immediately. All the samples were centrifuged at 3000 rpm for 10 minutes within 6 hours. After centrifugation, plasma was collected, frozen in Eppendorf (EP) tube and stored in a −80°C refrigerator. The blood samples were centrifuged at 3000 rpm for 10 minutes. Samples of sera were stored at –80°C until biochemical analysis.

### 2.5 Enzyme linked immunosorbent assay (ELISA)

ELISA kits were used for the protein detection assays to determine intracoronary and peripheral blood levels of human TL1A, hs-CRP, MPO, and IL-10. Protocols were conducted in accordance with the manufacturer's instructions, and results were recorded at 450 nm on an ELISA plate reader. The TL1A levels were compared and the correlation of TL1A with hs-CRP, MPO, and IL-10 was analyzed.

### 2.6 Follow-up and main outcome measures

Patients were followed after enrollment for 26.3 months via phone and at the outpatient department to record main adverse cardiac events (including cardiovascular death, definite or probable stent thrombosis, spontaneous myocardial infarction, or target vessel revascularization). The incidence rates of MACE were compared and the correlation of TL1A with MACE was analyzed.

### 2.7 Statistical analysis

Results are expressed as mean and standard deviation (SD). Comparisons between two groups and among multiple groups were conducted by Student *t* test and one-way ANOVA, respectively. ∗*P* < .05 and ∗*P* < .01 is considered significant. Two-variable correlation was analyzed by Pearson linear regression coefficient using IBM SPSS Statistics 21.

## 3 Results

### 3.1 Analysis of clinical baseline data

Baseline statistical analysis showed no significant difference among the groups (See Table 1 for details). Experimental results showed no significant differences in the body mass index, blood lipids (alanine aminotransferase (ALT), aspartate aminotransferase (AST), and serum creatinine (SCr)), and brain natriuretic peptides (BNP) (a heart failure indicator). Fisher exact test also shows that there is no significant difference among all the groups at the baseline (Table 2).

**Table 1:**
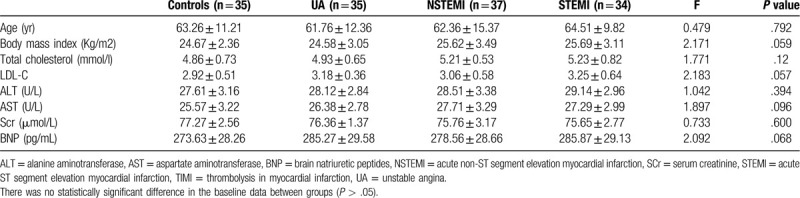
Comparison of CHD patients and healthy control individuals’ baseline data (quantitative indicators).

**Table 2:**
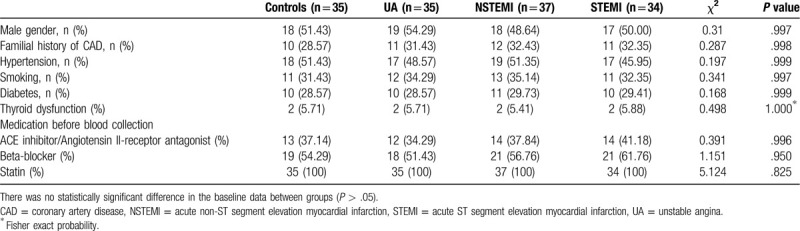
Comparison of clinical information of different groups.

### 3.2 Presence of slow coronary flow / no coronary reflow in NSTEMI and STEMI

The incidence rates of slow coronary flow/no coronary reflow were not identical among the four groups, showing statistically significant difference (Table 3). Compared with the control group, the incidence rate of slow coronary flow / no coronary reflow was higher in the NSTEMI and STEMI groups, showing statistically significant difference (the Fisher exact probability of 0.012 and 0.002 respectively). In addition, both NSTEMI and STEMI groups showed longer acute chest pain time and higher cTnI levels, as compared to control group (Table 4). Mann-Whitney test was performed to analyze the results of thrombus burden (Table 5). Compared with the control group, the thrombus burden in the STEMI group and NSTEMI group showed statistically significant difference (*P* < .05). The thrombus burden was more severe in the STEMI group than in the NSTEMI group (*P* < .05). Patients with TIMI Thrombus Grade 5 accounted for 52.94% of the emergency coronary intervention subgroup in the STEMI group (the highest proportion). Compared with the UA group, the thrombus burden was higher in the STEMI and NSTEMI group (*P* < .05).

**Table 3:**
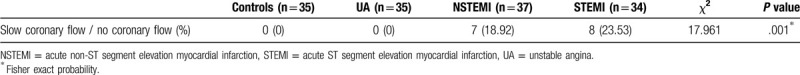
Comparison of slow coronary flow / no coronary reflow.

**Table 4:**
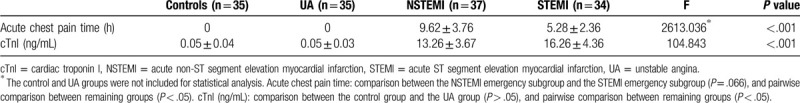
Comparison of acute chest pain time and cTnI levels.

**Table 5:**
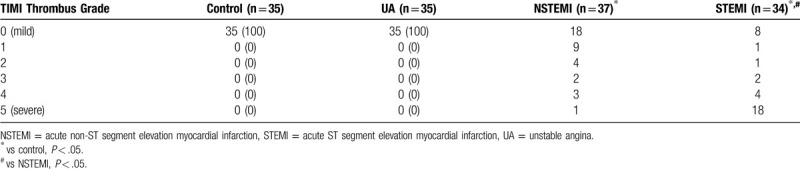
Comparison of thrombus burden (TIMI Thrombus Grade).

### 3.3 Levels of TL1A and other inflammatory factors in peripheral and coronary blood were higher in STEMI patients.

Compared with the control group, the TL1A levels in the coronary and peripheral blood were higher in both the STEMI and NSTEMI group, showing statistically significant difference (*P* < .05) (Table 6). In the STEMI and NSTEMI groups, the TL1A levels were higher in the coronary blood than in the peripheral blood, showing statistically significant difference (F = 1047.666 and 533.258 respectively, both *P* < .05). Furthermore, the TL1A levels in the coronary and peripheral blood were highly correlated with the correlation coefficient of 0.899, showing statistically significant difference (*P* < .001).

**Table 6:**
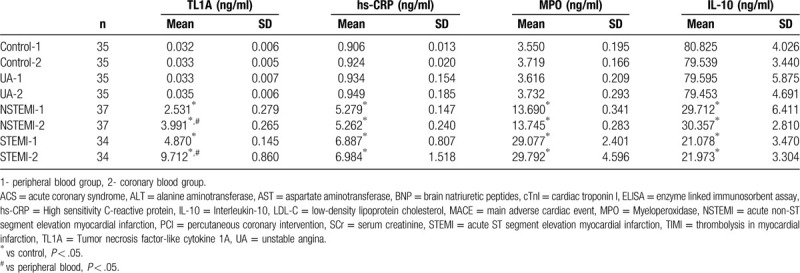
TL1A, hs-CRP, MPO, and IL-10 expression levels in CHD patients.

Moreover, the levels of hs-CRP and MPO in the coronary and peripheral blood were higher in the STEMI and NSTEMI groups as compared with the control group (*P* < .05). The level of IL-10 (an anti-inflammatory factor) in the coronary and peripheral blood were low in the STEMI and NSTEMI groups as compared with the control group (*P* < .05).

### 3.4 TL1A level was not related to Syntax score

Syntax score is an approach for risk stratification based on the anatomical features of coronary artery lesions (such as location, severity, bifurcation, calcification, etc.), and is used to quantitatively evaluate the complexity. There was no correlation between Syntax score and TL1A levels based upon correlation analysis of plasma TL1A levels in the high, intermediate, and low Syntax score groups (Table 7).

**Table 7:**
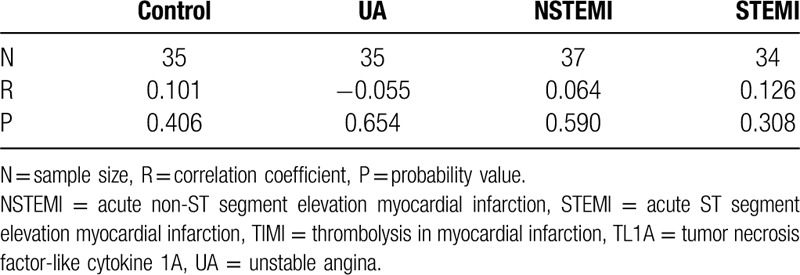
Correlation of plasma levels of TL1A with Syntax score.

### 3.5 High TL1A level was correlated to main adverse cardiac event (MACE)

Eleven patients were lost to follow up, including 2 in the control group, 2 in the UA group, 2 in the NSTEMI group, 2 in the STEMI group. The follow-up rate was 94.77% and the median follow-up time was 26.3 months. In the NSTEMI and STEMI with high TL1A expression levels, the incidence rate of MACE was 5.71%, 6.25% respectively (Table 8). And the Spearman correlation test shows that the correlation coefficient between the incidence of MACE and the TL1A levels was 0.143, showing statistically significant difference (*P* = .037).

**Table 8:**
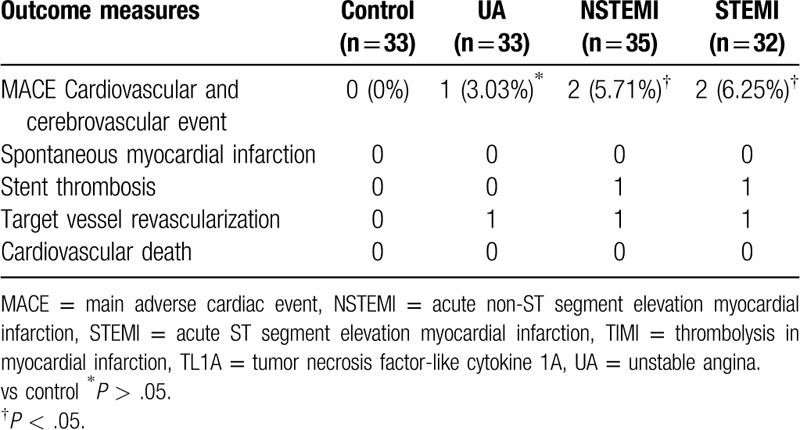
Follow-up results of MACE.

### 3.6 Correlation of TL1A with other risk factors

Regardless of the data distribution type, Spearman correlation test indicated that the level of TL1A was positively correlated with the level of cTnI, coronary thrombus burden, slow coronary flow / no coronary reflow, hs-CRP and MPO, but negatively correlated with the IL-10 level, showing statistically significant difference (Table 9). Multivariate regression analysis indicates that TL1A was a most significant predictor for MACE in NSTEMI and STEMI patients (Table 10).

**Table 9:**
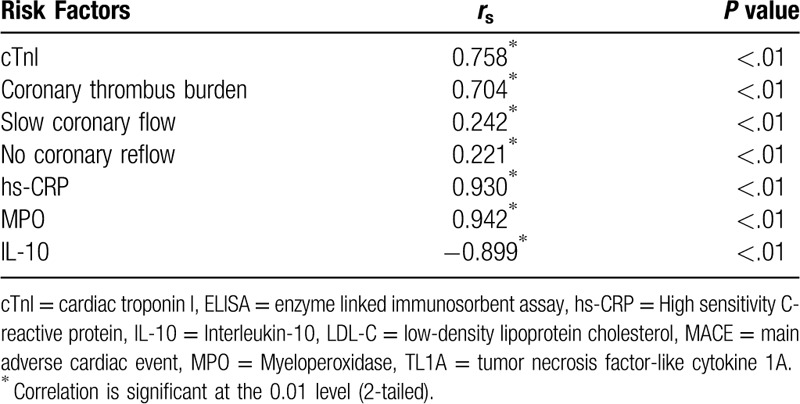
Correlation of TL1A with other risk factors.

**Table 10:**
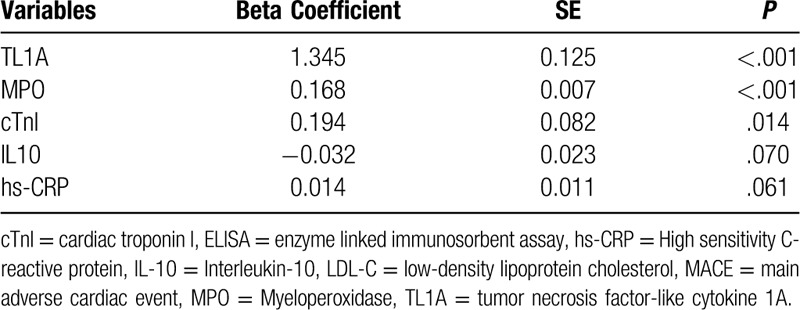
Multivariable analysis of TL1A and other factors associated with MACE.

## 4 Discussion

Acute coronary syndrome (ACS) is induced by myocardial ischemia and hypoxia resulting in atherosclerosis and lumen stenosis. At present, it is believed that ACS is an inflammatory condition, and development involves a variety of inflammatory cytokines and immune cells. TL1A is a newly discovered cytokine belonging to the tumor necrosis factor superfamily 15 that exists in two forms (membrane-bound and secreted form) to exert its biological functions. Studies have shown that TL1A can induce the expression of cytokines and chemokines, promote extracellular matrix degradation enzymes, and decrease the stability of plaques.^[13]^ Yoon-Joong Kang et al also found that macrophages and foam cells^[9]^ are positive with high expression levels of TL1A in human carotid atherosclerotic plaques.

TL1A expression is increased in both plasma and inflamed tissues in autoimmune diseases.^[14–17]^ TL1A expression is inducible by TNF and IL-1α.^[5,18]^ TL1A induces inflammation and apoptosis in DR3-expressing cell lines. In T cells, TL1A acts as a co-stimulator that increases IL-2 responsiveness and secretion of pro-inflammatory cytokines both in vitro and in vivo. Research studies have also confirmed that TL1A can act as a pleiotropic immunomodulator for enhancing angiogenesis, which may be associated with the progression of ACS.

However, it has not been reported whether the expression levels of TL1A in patients with ACS are elevated, whether there are differences in the expression of TL1A in the UA, NSTEMI, and STEMI groups, or whether there is any change in the concentration of circulating TL1A locally in the coronary ruptured plaque. In this study, the correlation of TL1A levels with the prognosis of MACE was further analyzed during the follow-up period to evaluate whether TL1A is of clinical predictive significance. Thus, changes in TL1A expression levels were analyzed in coronary and peripheral blood of groups in acute phase within 12 hours of ACS attack.

As the major therapy of acute myocardial infarction, PCI plays an important role in opening the occluded coronary artery, reducing the myocardial infarction size, and improving the clinical prognosis for patients. However, between 20% and 30% of patients have slow flow / no reflow (NR) after coronary stent revascularization, which lowers the therapeutic value of coronary intervention and increases the incidence of coronary thrombosis, heart failure, and cardiovascular death significantly, hence seriously affecting their prognosis.^[19–21]^ Our findings suggested that the incidence rate of slow coronary flow / no coronary reflow was significantly higher in the NSTEMI and STEMI groups when compared with the control group. These results may be associated with severe coronary stenosis and significant thrombus burden in the STEMI and NSTEMI emergency subgroups, resulting in significantly slowed coronary blood flow or no perfusion of coronary blood flow.^[22]^ There was no significant difference in cTnI levels between the UA group and the control group. Pairwise comparison between remaining groups showed all *P* < .05, which was possibly related to the time of myocardial infarction and the extent of infarction.

*ST segment elevation* is associated with ischemic myocardial injury or necrosis of the myocardium *resulting in complete occlusion of a major coronary artery due to* platelet aggregation and thrombosis of the infarct-related artery. Coronary angiography was performed in all patients in this study. The criteria for judging thrombosis are that the filling defect present in multiple cardiac cycles is visible in the lumen before and after the guide wire passes through the lesion and at multiple angles during angiography, and that the intimal dissection in the false lumen caused by the guide wire is excluded. The TIMI thrombus burden classification was defined as follows: in TIMI thrombus grade 0, no angiographic evidence of thrombus; in TIMI thrombus grade 1, angiographic features suggestive of thrombus such as haziness of contrast; in TIMI thrombus grade 2, definite thrombus present in multiple angiographic projections (the greatest dimension of thrombus is <1/2 vessel diameter); in TIMI thrombus grade 3, definite thrombus appears in multiple angiographic views (great dimension from >1/2 to <2 vessel diameters); in TIMI thrombus grade 4, definite large size thrombus present (greatest dimension >2 vessel diameter); and in TIMI thrombus grade 5, definite complete thrombotic occlusion of a vessel.^[23]^ Comparison of the thrombus burden showed significant differences between the groups and the control except for the UA group, and that the STEMI group had the heaviest thrombus burden and the highest proportion of TIMI Thrombus Grade 5 (52.94%). A large number of studies have confirmed that thrombus is a high risk factor of PCI, which is an important contributor to poor myocardial perfusion or no reflow after surgery.^[24]^

TL1A is an inflammatory factor expressed in carotid plaques and may be involved in the formation and progression of atherosclerosis. However, there have been no reports on the expression of TL1A in the blood circulation of ACS and other acute coronary syndromes (including UA, NSTEMI and STEMI), especially in the local blood circulation of coronary plaques. The purpose of this study was to investigate the role of TL1A in local and systemic blood circulation of ACS patients by collecting the coronary and peripheral blood samples of all groups to detect the TLIA levels and expression levels of hs-CRP and MPO (recognized pro-inflammatory factors) as well as IL-10 (an anti-inflammatory factor). Analysis of results showed that TLIA levels in the coronary and peripheral blood and plasma of acute myocardial infarction patients (emergency and selective subgroups) were higher than the control group, showing a statistically significant difference. TLIA levels in the coronary and peripheral blood and plasma of the UA group were higher than the control group, showing no statistically significant difference, among which the TLIA levels were highest in the coronary blood of the STEMI group. The correlation analysis of TL1A levels in and out of coronary artery indicated that coronary TL1A level was highly correlated with peripheral TL1A level and the levels of TL1A in coronary thrombus and local plaques could be reflected by taking plasma from the peripheral blood. Meanwhile, the correlation analysis also showed that the level of TL1A was positively correlated with slow coronary flow/no coronary reflow and coronary thrombus burden, suggesting that TL1A plays an important role in both the rupture of local atherosclerotic plaques and the thrombosis in the coronary artery.

By detecting hs-CRP, MPO, and IL-10 simultaneously, it was found that in the UA, NSTEMI, and STEMI groups, the plasma hs-CRP and MPO levels increased while the plasma IL-10 levels decreased, showing hs-CRP and MPO are recognized pro-inflammatory factors^[25–27]^ and IL-10 is a definite anti-inflammatory factor.^[28]^ The correlation analysis also showed that TL1A was positively correlated with hs-CRP and MPO, but negatively correlated with IL-10, suggesting that TL1A may lead to the occurrence of coronary events by promoting inflammatory-related mechanisms. In addition, there was a positive correlation between TL1A levels and cTnI levels, suggesting that TL1A levels could reflect the degree and extent of myocardial infarction. cTnI is a marker for detection of myocardial damage and has been widely used to predict acute myocardial infarction or death in patients with unstable coronary heart disease. TL1A is correlated with endothelial dysfunction and atheromatic plaque formation and can be used to predict the prognosis of both ACS and stable coronary heart disease.^[29]^ TL1A was also positively correlated with slow coronary flow and no coronary reflow, which had a high incidence rates in the NSTEMI and STEMI groups, suggesting that TL1A was involved in the occurrence of adverse events of coronary perfusion and played an important role in coronary endothelial dysfunction.

Syntax score is intended to be a preliminary means for choosing an appropriate surgical approach^[30,31]^ and can guide the choice of therapies. Previous studies have demonstrated plasma concentration levels of TL1A may predict coronary artery disease severity.^[32]^ There was no correlation between Syntax score and TL1A levels based upon correlation analysis. However, the plasma TL1A level was positively correlated with troponin I level, coronary thrombus burden, and slow coronary flow/no coronary reflow. All of these indicators are pathophysiological factors in the acute phase of coronary atherosclerotic heart disease, suggesting that TL1A is an acute inflammatory factor, especially in unstable or ruptured plaques, but only anatomical-morphological changes of coronary atherosclerosis could not lead to the changes of plasma TL1A levels in stable plaques. Taken together, our results suggest TL1A expression levels can predict the acute events of ACS.

During the experiment, patients were followed up for 26.3 months. In the NSTEMI, STEMI groups with high TL1A expression levels, the incidence rate of MACE was 5.71%, 6.25%, respectively. The Spearman correlation test suggested the correlation of the incidence of MACE with the TL1A levels. Both indicated that TL1A can be the MACE predictor.

Limitations of the present study: Although TL1A is pro-inflammatory factor, it may not fully reveal the ACS patients’ inflammation phrase. Other inflammation-associated biomarkers such as circulating microRNAs should be considered in further study. For instance, microRNA-16, which is closely linked to inflammation during peripheral ischemia and it is involved in vascular remodeling of infarcted heart,^[33,34]^ can serve as an additional biomarker with TL1A to predict the prognosis of ACS. Another limitation of the study is the relatively small sample size in each group. To provide a more comprehensive understanding of TL1A in ACS, future studies will involve larger patient cohorts with longer follow-up term.

In summary, this study demonstrated that intracoronary and peripheral blood TL1A expression levels were upregulated in patients with acute ACS. Our findings indicate that TL1A may be used as an inflammatory factor to evaluate the recent onset of ACS, and can serve as a potential indicator for the long-term prognosis of ACS patients.

## Author contributions

XJC, YSG, LL and ZL designed the experiments. XJC and YSG performed the experiments and analysis. LL and SLZ helped analyze the data. XJC and LL wrote the manuscript. ZLL revised the manuscript. All authors read and approved the final manuscript.
