# Intracoronary and peripheral blood levels of TNF-like Cytokine 1A (TL1A) in patients with acute coronary syndrome: Erratum

**DOI:** 10.1097/MD.0000000000021285

**Published:** 2020-07-10

In the article, “Intracoronary and peripheral blood levels of TNF-like Cytokine 1A (TL1A) in patients with acute coronary syndrome”,^[1]^ which appears in Volume 99, Issue 22 of *Medicine*, the author list appeared incorrectly as Xinjing Chen, Yansong Guo, and Shengli Zhang. The author list has since been corrected to Xinjing Chen, Yansong Guo, Shengli Zhang, and Zhiliang Li. The Zhiliang Li has been added as a corresponding issue. The author contribution's first sentence has also been updated from “XJC, YSG and LL designed the experiments” to “XJC, YSG, LL, and ZL designed the experiments.”
